# Toll-Like Receptor 8 Is a Major Sensor of Group B *Streptococcus* But Not *Escherichia coli* in Human Primary Monocytes and Macrophages

**DOI:** 10.3389/fimmu.2017.01243

**Published:** 2017-10-03

**Authors:** Birgitta Ehrnström, Kai Sandvold Beckwith, Mariia Yurchenko, Siv Helen Moen, June Frengen Kojen, Germana Lentini, Giuseppe Teti, Jan Kristian Damås, Terje Espevik, Jørgen Stenvik

**Affiliations:** ^1^Centre of Molecular Inflammation Research, Department of Cancer Research and Molecular Medicine, Norwegian University of Science and Technology, Trondheim, Norway; ^2^Department of Infectious Diseases, Clinic of Medicine, St. Olavs Hospital HF, Trondheim University Hospital, Trondheim, Norway; ^3^Department of Clinical and Experimental Medicine, University of Messina, Messina, Italy

**Keywords:** infection, inflammation, pattern recognition receptors, human TLR8, primary human phagocytes

## Abstract

TLR8 is the major endosomal sensor of degraded RNA in human monocytes and macrophages. It has been implicated in the sensing of viruses and more recently also bacteria. We previously identified a TLR8-IFN regulatory factor 5 (IRF5) signaling pathway that mediates IFNβ and interleukin-12 (IL-12) induction by *Staphylococcus aureus* and is antagonized by TLR2. The relative importance of TLR8 for the sensing of various bacterial species is however still unclear. We here compared the role of TLR8 and IRF5 for the sensing of Group B *Streptococcus* (GBS), *S. aureus*, and *Escherichia coli* in human primary monocytes and monocyte-derived macrophages (MDM). GBS induced stronger IFNβ and TNF production as well as IRF5 nuclear translocation compared to *S. aureus* grown to the stationary phase, while *S. aureus* in exponential growth appeared similarly potent to GBS. Cytokine induction in primary human monocytes by GBS was not dependent on hemolysins, and induction of IFNβ and IL-12 as well as IRF5 activation were reduced with TLR2 ligand costimulation. Heat inactivation of GBS reduced IRF5 and NF-kB translocation, while only the viable *E. coli* activated IRF5. The attenuated stimulation correlated with loss of bacterial RNA integrity. The *E. coli*-induced IRF5 translocation was not inhibited by TLR2 costimulation, suggesting that IRF5 was activated *via* a TLR8-independent mechanism. Gene silencing of MDM using siRNA revealed that GBS-induced IFNβ, IL-12-p35, and TNF production was dependent on TLR8 and IRF5. In contrast, cytokine induction by *E. coli* was TLR8 independent but still partly dependent on IRF5. We conclude that TLR8-IRF5 signaling is more important for the sensing of GBS than for stationary grown *S. aureus* in human primary monocytes and MDM, likely due to reduced resistance of GBS to phagosomal degradation and to a lower production of TLR2 activating lipoproteins. TLR8 does not sense viable *E. coli*, while IRF5 still contributes to *E. coli*-induced cytokine production, possibly *via* a cytosolic nucleic acid sensing mechanism.

## Introduction

The Gram-positive bacterium *Streptococcus agalactiae* [Group B *Streptococcus* (GBS)] is an asymptomatic colonizer of the intestinal and genital tract of 15–30% adults and part of the human microbiota (1). But GBS also has a potent invasive potential, triggers excessive inflammation, and is a leading bacterial agent in neonatal infections causing sepsis, pneumonia, and meningitis. In adults, GBS is an emerging cause of life-threatening infections (2, 3). The normal physiological function of inflammation is to promote innate and adaptive defense mechanisms. However, in the acute phase of sepsis excessive inflammation can lead to shock and organ dysfunction, while more prolonged stages can be characterized by suppression of phagocyte and lymphocyte functions, impaired immunity, and secondary infections (4). GBS, *Staphylococcus aureus*, and *Escherichia coli* are major human pathogens. Protection against these infections depends on TLR- and interleukin-1 receptor (IL-1R) signaling, and susceptibility is high in humans with genetic deficiency in the downstream signaling components myeloid differentiation factor 88 (MyD88) or IL-1R-associated kinase 4 (IRAK-4), especially before adulthood (5). Clarification of the innate sensing mechanisms is essential to understand their roles in maladaptive and protective immune reactions in innate and adaptive immunity and to identify new targets for treatment of invasive infections.

Innate immune cell sensing of GBS *via* pattern recognition receptors (PRRs) and the subsequent inflammatory reactions have mainly been characterized using the murine model system. This revealed a strong dependency on MyD88 *in vitro* and *in vivo* and was interpreted as evidence of TLR-mediated recognition of the entire heat-inactivated (HI) GBS bacteria, although the proinflammatory responses were mainly independent of TLR2, TLR4, and TLR9 (6). Still, at least some GBS strains release lipoproteins which can activate macrophages *via* TLR2/6 and mediate protection in a low-dose model of GBS sepsis (7). In murine conventional dendritic cells (cDC) GBS-induced IFNβ production is TLR7 dependent, suggesting that ssRNA can be an important pathogen-associated molecular pattern (PAMP) sensed by innate immune cells (8). In line with this, depletion of ssRNA from HI GBS and other gram-positive bacteria, but not gram-negative species, reduced the cytokine induction by murine macrophages and human PBMCs. Moreover, the responses in murine cells are MyD88 and UNC-93B dependent (9), and TLR13 is subsequently identified as an endosomal sensor of bacterial 23S rRNA in rodents (10, 11). TLR13 contributes to TNF induction by HI GBS in cDC and macrophages, but for the sensing of viable GBS TLR13 appears redundant (12). A recent study suggests that TLR13 is the major PRR of GBS in various types of murine tissue macrophages, but not in murine monocytes or in skin infection models where MyD88-dependent but UNC-93B-independent mechanisms dominate (13). Mouse models also show a critical role of IL-1β for resistance against acute GBS infections, likely due to the essential role of neutrophil recruitment to the site of infection. IL-1β production in murine GBS infection relies not only on pro-IL-1β production by endosomal TLR, but also on hemolysin dependent release of GBS RNA into the cytosol which triggers NLRP3-activation and pro-IL-1β cleavage, a process occurring in both macrophages, DC, and neutrophils (14–16).

Humans do not express TLR13, but in contrast to mice human myeloid cells express TLR8 as a functional sensor of uridine-rich ssRNA (17–19). It was recently clarified that TLR8 is a sensor of the RNA degradation products uridine and short ssRNA oligomers which cooperatively bind to and activate the preformed TLR8 dimer (20, 21). While human TLR8 can sense RNA of viruses including HIV (22, 23), its physiological role has remained unclear, partly because of the lack of a small-animal model and cellular tools. We and others recently showed that TLR8 contributes to the sensing of entire bacteria including *S. aureus, Streptococcus pyogenes* (Group A *Streptococcus*), and *E. coli* (19, 24, 25). We specifically found that TLR8 induced IFNβ and IL-12 production upon *S. aureus* infection of primary monocytes and monocyte-derived macrophages (MDM) *via* a TAK1-IkB kinase (IKKβ)-IFN regulatory factor 5 (IRF5) signaling pathway. We also discovered that TLR2 signaling antagonized TLR8-mediated IRF5 activation and IFNβ and IL-12 production (24).

Here, we identify TLR8 as a major sensor of GBS in human monocytes and MDM. TLR8 is critical for the activation of IRF5 and the induction of IFNβ and IL-12 production by GBS and contributes to TNF induction. In contrast, TLR8 appears less important for detecting *S. aureus* in stationary growth phase, and TLR8 does not mediate responses to *E. coli*.

## Materials and Methods

### Materials

Concentrations of IFNβ were determined with the VeriKine-HS human IFNβ serum ELISA kit (PBL Assay Science, Piscataway, NJ, USA). TNF and IL-6 ELISA duo-kits were from R&D Systems, and BioPlex assays were from Bio-Rad. The cytokine levels were determined as per the manufacturer’s instructions. The PRR ligands FSL1, CL75, LPS O111:B4, poly(deoxyadenylic-deoxythymidylic) acid (poly-dA:dT; B-DNA), polyinosinic-polycytidylic acid [poly(I:C)], and polyuridylic acid (pU) were purchased from Inviviogen. Poly-l-arginine (pLA) was from Sigma. Lipofectamine 2000 (L2K) was purchased from Invitrogen.

### Bacteria and Bacterial RNA Isolation

*Staphylococcus aureus* 113 strain and its isogenc *lgt* mutant strain (Δ*lgt*) were generously provided by prof. Friedrich Göetz (University of Tübingen, Tübingen, Germany), while the Cowan strain was generously provided by Prof. Timothy Foster (Trinity College, Dublin, Ireland). The GBS NEM316 wild type (wt) and its isogenic *lgt* mutant strain (Δ*lgt*) were generously provided by prof. Philipp Henneke (University of Freiburg, Germany). The GBS NEM316 β-hemolysin/Christie Atkins Munc-Petersen (CAMP)-deficient strains (Δ*cylE*, Δ*cfb*, and double deficient Δ*cylE*Δ*cfb*) have previously been described (26). The *E. coli* Seattle 1946-strain was purchased from the American Type Culture Collection (ATCC 25922).

*E. coli* and GBS were grown on tryptic soy agar while *S. aureus* strains were grown on mannitol salt agar. To prepare bacteria for use in infection experiments, colonies were picked and grown in 5 ml tryptic soy broth (*E. coli* and *S. aureus*) or Todd-Hewitt broth (GBS) during vigorously shaking at 37°C overnight (12–18 h). HI was done at 70°C for 30 min. To quantify the bacteria, we initially determined the concentrations of GBS, *S. aureus*, and *E. coli* in cultures (stationary phase) by manual microscopy counting, by using a Neubauer Improved counting chamber with 0.02 mm depth (Assistant, Germany) and a Nikon Eclipse E100 phase-contrast microscope with Plan 100× oil objective (NA 1.25). We subsequently determined the OD600 of the cultures using a cell density meter (Ultrospec 10, Amersham Biosciences) for multiple dilutions of each culture within an OD600 range of 0.05–1.20. Standard curves for OD600 versus the corresponding bacteria concentrations were made, and were close to linear in the examined range. An OD600 of 1.00 on the specified equipment correspond to 5.6 × 10^8^/ml of *E. coli* and 1.5 × 10^9^/ml of GBS and *S. aureus*. In subsequent experiments the OD600 of the bacterial cultures were used to calculate the bacteria concentration using the standard curves. To calculate the MOI, the bacterial concentrations were converted to CFU, and blood agar counting revealed bacteria-to-CFU ratio of 1:1 for *E. coli*, 5:1 for *S. aureus*, and 10:1 for GBS, which reflects that *S. aureus* and GBS grow in clumps and strings, respectively. The method of quantification was validated for bacterial cultures in both stationary and exponential phases of growth.

Bacterial RNA extraction was conducted in accordance with the procedure described (15) with some modifications. Briefly, bacterial cell extracts were obtained by vortexing both live and HI GBS (grown in the stationary phase) on ice in the presence of glass beads (0.5 mm, Precyllys). Total RNA was purified by the RNeasy Mini kit using DNAse (Qiagen), according to the manufacturer’s protocol. The RNA integrity was examined using Agilent 2100 bioanalyzer (Agilent Technologies, Inc.), and the concentrations were determined with Qbit Fluorometer and Nanodrop (Thermo Fisher Scientific).

### Blood, Monocytes, and Stimulation/Infection

Human PBMCs were isolated from buffycoats using Lymphoprep (Axis-Shield) as described by the manufacturer. Monocytes were purified by adherence in culture plates or by negative selection using magnetic beads (Miltenyi Biotec Pan Monocyte Isolation Kit). Monocytes were maintained in RPMI 1640 (Life Technologies) supplemented with 10% pooled human serum.

For infection of monocytes and macrophages, extracellular bacteria were killed after 1 h by addition of gentamicin to 100 μg/ml final concentration.

### Macrophages

Monocyte-derived macrophages were generated by differentiation of monocytes in RPMI 1640 with 300 μl 30% pooled human serum for 5–6 days in 96-Well Glass Bottom Microwell Plates (MGB096-1-2-LG-L, Matriplate) or μ-Plate 96 Well ibiTreat (Ibidi). Medium was replaced with RPMI 1640 containing 10% human serum before infection, stimulation, or siRNA treatment.

TLR8 knockout THP1 monocytes (19) were generously provided by prof. Veit Hornung (University of Bonn, Germany). A fluorescent TLR8 construct TLR8-mNeonGreen was made by Gateway cloning. hTLR8 isoform 1 and mNeonGreen (for fluorescent construct) were amplified by PCR from pUno-hTLR8a (Invivogen) and pNCS-mNeonGreen (Allele Biotech), respectively. For non-fluorescent constructs the following PCR primers were used.

TLR8-fw: GGGGACAAGTTTGTACAAAAAAGCAGGCTT CATGGAAAACATGTTCCTTCAG, TLR8-rw: GGGGACCACT TTGTACAAGAAAGCTGGGTTTTAGTATTGC TTAATGGAATCG. For fluorescent construct, the fragments were BP recombined with pDONR221 L1-R5 and L5-L2 vectors to generate multisite pEntry clones. A lentiviral expression clone was generated by LR recombination between pEntry-TLR8-L1-R5, pEntry-mNeonGreen-L5-L2 and pLenti-CMV-Puro-DEST (w118-1) (a gift from Eric Campeau, Addgene plasmid # 17452), giving a C-terminal in-frame fusion of TLR8 to mNeonGreen. For non-fluorescent TLR8 expressing construct, fragment was BP recombined with pDONR221 vector followed by LR recombination with pLenti-CMV-Puro-DEST (w118-1) vector. All constructs were verified by Sanger sequencing (GATC biotech). Lentiviral particles were produced in HEK293T cells using a third generation lentiviral packaging system (Addgene plasmids # 12253, 12251, and 12259) and concentrated using PEG precipitation as described elsewhere (27). For fluorescent construct, TLR8 knockout THP1 cells were spin-transduced in the presence of 8 µg/mL polybrene and 1 mg/ml Synperonic-F108 (Sigma) (28), selected on 0.6 µg/ml puromycin and subjected to one round of FACS to select for a moderate expression level. For non-fluorescent TLR8 overexpression, THP1 WT cells (ATCC) were transduced by viral particles containing a pLenti-CMV-Puro-DEST-TLR8 construct, or with virus containing empty vector (for control cell line). Cells were grown for selection on 1 µg/ml puromycin for 4 weeks. THP1 cells were differentiated using 100 ng/ml phorbol 12-myristate 13-acetate [PMA (Sigma)] for 3 days, followed by 1 day rest in fresh media before infection.

### Western Blotting

Cells were lysed in lysis buffer and denatured in 1× NuPage LDS (Invitrogen) sample buffer supplemented with 25 mM DTT for 10 min at 70°C. The samples were separated on 4–12% Bis-Tris polyacrylamide gel and transferred to a nitrocellulose membrane using the iBlot Dry Blotting System (Invitrogen). The blots were incubated with horseradish peroxidase-conjugated immunoglobulin’s (DAKO) and developed with Super Signal West Femto Maximum Sensitivity Substrate (Thermo Scientific) before images were obtained with Odyssey Fc Imaging System (LI-COR). TLR8 overexpression in THP-1 subclones was confirmed by Western blotting using rabbit antihuman TLR8 XP mAb [Cell Signaling Technology (CST), no. D3Z6J]. Activation of IRF3 was done using rabbit antiphospho-IRF3 mAb (CST, no. D6O1M). Anti GAPDH (Abcam, #ab8245) served as a loading control.

### Silencing and Quantitative Real-time PCR (qPCR)

A pool of four individual ON-TARGETplus siRNAs (Dharmacon) was transiently transfected using siLentFect (Bio-Rad), yielding a final concentration of 5 nM siRNA. The transfection was repeated after 3 days, and the silenced MDM were infected with bacteria or stimulated with ligands for 4 h. RNA was isolated with an RNeasy 96 Plus kit (Qiagen), cDNA was transcribed with a Maxima cDNA synthesis kit (Thermo Fisher Scientific), and relative quantification by qPCR was done with StepOnePlus using TaqMan probes (Life Technologies) and Perfecta qPCR FastMix from Quanta. Probes used were: IFNβ, Hs01077958_s1; TNF, Hs00174128_m1; IL-6 Hs00985639_m1; IL-12A Hs1073447_m1; TBP, Hs00427620_m1, IKKβ Hs00233287_m1, cGAS/MB21D1 Hs00403553_m1, MyD88 Hs00182082_m1, STING/TMEM173 Hs00736958_m1, TLR7 Hs00152971_m1, TLR8 Hs00607866_mH, IRF5 Hs00158114_m1, and TBK1 Hs00179410_m1. TBP served as endogenous control, and relative expression was calculated as fold induction by stimulation or infection.

### Immunofluorescence (IF) and Scan^R Analyses

Immunofluorescence staining of TLR8 and transcription factors and quantification of nuclear accumulation by high content screening (Scan^R system, Olympus) was done as previously described (24). The following antibodies were used: mouse antihuman IRF5 mAb (Abcam, #10T1), rabbit antihuman IRF3 XP mAb [Cell Signaling Technology (CST), no. 11904], rabbit antihuman p65/RelA XP mAb (CST, no. 8242), rabbit antihuman p65 A (Santa Cruz Biotechnology, no. sc-109), rabbit antihuman IRF1 XP mAb (CST, no. 8478), and rabbit antihuman TLR8 XP mAb (CST, no. D3Z6J). For TLR4 IF staining monocytes were washed by ice-cold PBS twice, fixed by cold methanol-acetone (1:1) at −20°C overnight, rehydrated in PBS for 1 h, and blocked by 20% human serum in PBS for 30 min. After blocking, cells were incubated with primary antibodies diluted in 2% human serum [2 µg/ml rabbit anti-TLR4 IgG (H-80) or normal rabbit IgG (Santa Cruz Biotechnology)] at +4°C overnight, washed three times by PBS with 2% human serum, incubated with secondary A488-labeled goat antirabbit IgG (Life Technologies). After three washes, cells were left in PBS at +4°C prior to confocal microscopy imaging, which was done using Leica SP8 with a 63× objective (NA 1.4).

### Statistical Analyses

Statistical analyses were done on data merged from independent experiments (not replicates). The number of independent experiments (*n*) is given in the figure legends. Prior to statistical analyses, the data were log transformed to increase the likelihood of a Gaussian distribution. Significant differences was tested by one-way RM ANOVA with Dunette’s posttest, or by two-way RM ANOVA with Bonferroni multiple comparison posttest. Significance levels: *p < 0.05, **p < 0.01, and ***p < 0.001. All analyses were performed with GraphPad Prism v5.03.

### Safety

The research was carried out according to the standard institutional safety procedures (biosafety level II) at the Norwegian University of Science and Technology.

## Results

### GBS Elicits Stronger Induction of IFNβ and TNF Production Compared to Stationary Grown *S. aureus*

**Figure 1:**
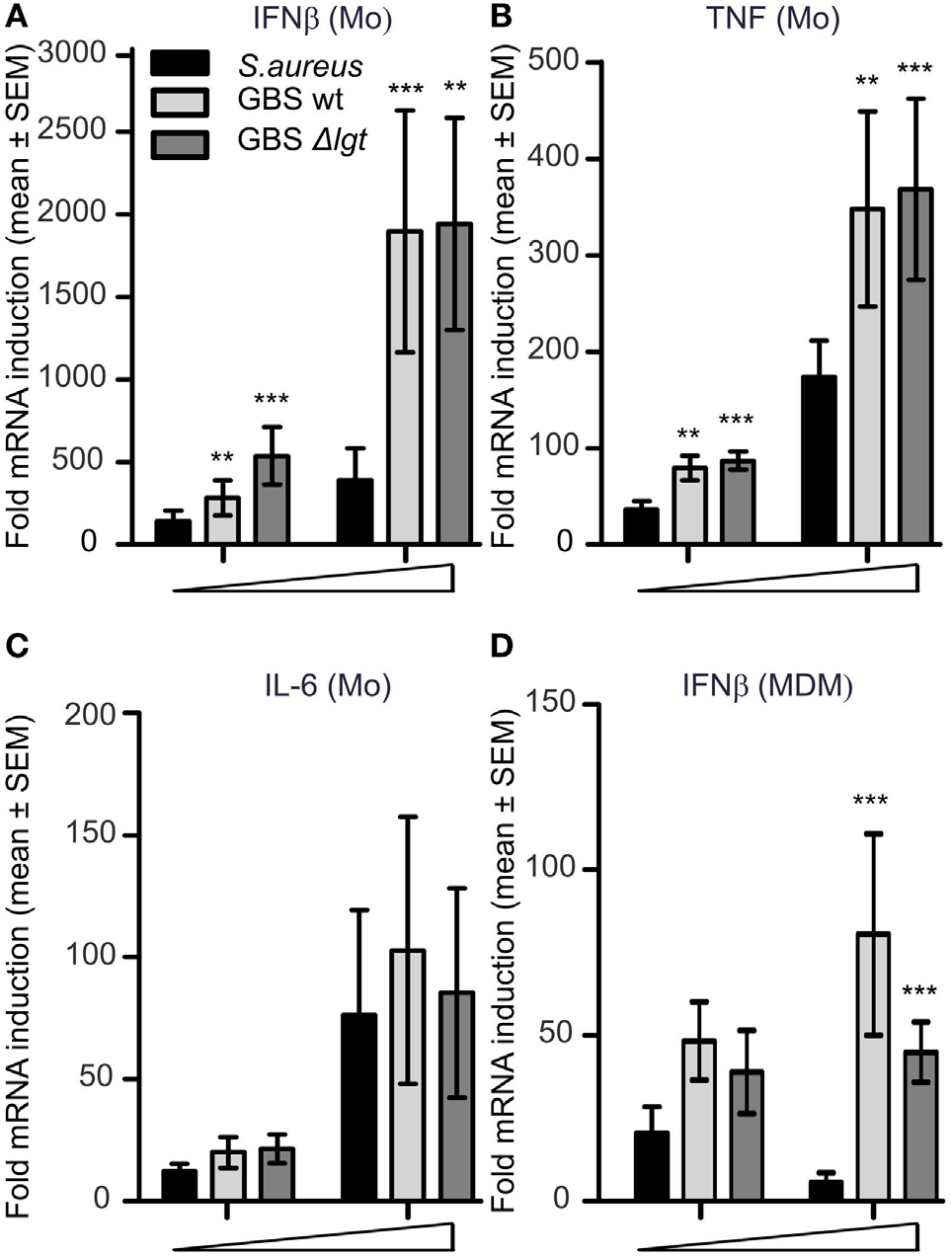
Group B *Streptococcus* (GBS) induce IFNβ and TNF *via* a TLR2-independent mechanism and is a more potent trigger than *Staphylococcus aureus* from stationary growth. Human primary monocytes (Mo) and monocyte-derived macrophages (MDM) were infected with viable GBS wt, lipoprotein diacylglyceryl transferase deficient GBS (Δ*lgt*) and *S. aureus* 113-strain. Cytokine induction was determined by quantitative real-time PCR (qPCR) 3 h postinfection in monocytes **(A–C)** [multiplicity of infection (MOI): 0.4–4.0 for *S. aureus*, and 0.2–2.0 for GBS], or 4 h postinfection in MDM **(D)** (MOI 4.0–40 for *S. aureus* and 2.0–20 for GBS). Statistical significance was tested relative to *S. aureus. n* = 4.

We recently showed how IFNβ and IL-12-p70 production by human primary monocytes upon *S. aureus* infection is mediated by TLR8-IRF5 signaling (24). Significant reduction in TLR8-IRF5 activation is seen with *S. aureus* strains activating TLR2 and is also characteristic of *S. aureus* when it is grown to the stationary phase compared to the exponential phase (24). To investigate the role of TLR2 and growth phases on GBS induced cytokines, we infected primary human monocytes with viable GBS NEM316 strains (GBS wt) and its isogenic lipoprotein deficient strain (GBS Δ*lgt*) which does not activate TLR2 (7). *S. aureus* wt and *S. aureus* Δ*lgt* strains were included for comparison. With bacteria from the stationary growth phase both GBS wt and GBS Δ*lgt* induced more IFNβ and TNF than *S. aureus* wt strain (Figures 1A,B), while induction of IL-6 was not significantly different between the species (Figure 1C). There were no significant differences in cytokine levels induction by GBS wt and GBS Δ*lgt* strains, suggesting that GBS produced little TLR2-activating lipoproteins, or that the mechanism of cytokine induction by GBS was not affected by TLR2 costimulatory molecules. The low IFNβ and TNF induction by *S. aureus* wt and *S. aureus* Δ*lgt* compared to GBS was a characteristic of the stationary growth phase, as similar cytokine induction was for both species in the exponential growth phases (Figure S1 in Supplementary Material). The level of IFNβ production was in particular suppressed for *S. aureus* wt strain in stationary phase and when the bacterial culture media was included, suggesting both TLR2-mediated suppression of TLR8-IRF5 inhibition and growth-phase dependent changes of *S. aureus*, in agreement with our previous findings of Ref. (24). In contrast, GBS wt and GBS Δ*lgt* induced similar cytokine levels in either condition (Figure S1 in Supplementary Material). Infection of MDM showed similar differences between GBS and *S. aureus* for the induction of IFNβ, although the level of cytokine induction was lower (Figure 1D). Altogether, GBS induce more IFNβ and TNF compared to *S. aureus* in stationary growth phase, and GBS-mediated cytokine induction is mainly independent of TLR2 agonistic GBS lipoproteins.

### Induction of IFNβ by GBS in Human Monocytes Is Not Dependent on Hemolysins and Is Antagonized by TLR2 Ligand

**Figure 2:**
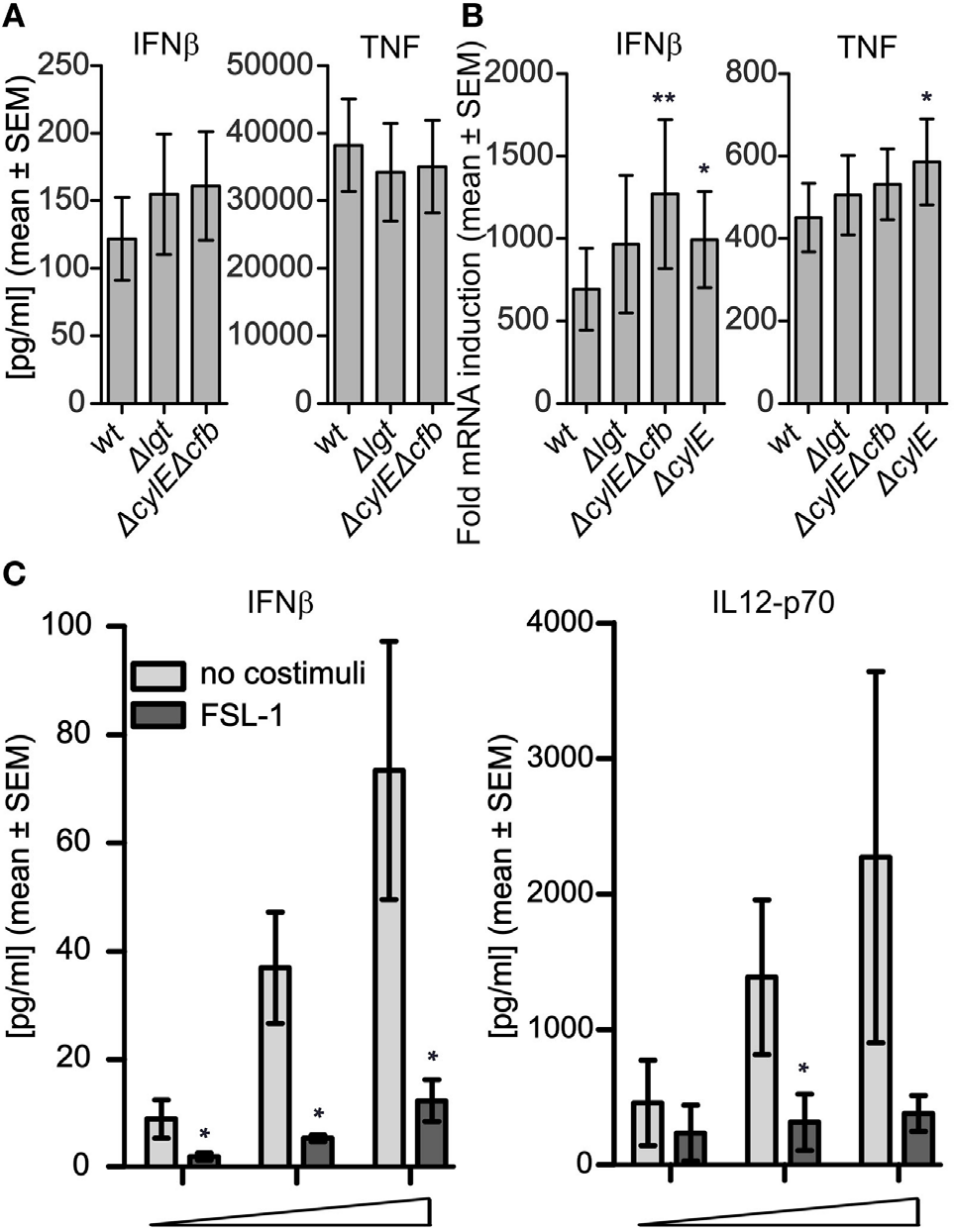
Group B *Streptococcus* (GBS)-induced production of IFNβ in monocytes is not dependent on hemolysin and is antagonized by TLR2 ligand. **(A)** Infection with viable GBS wt, GBS Δ*lgt*, and GBS hemolysin deficient strains (Δ*cylE*Δ*cfb*), which is deficient for both the β-hemolysin CylE gene (*cylE*) and the cohemolysin Christie Atkins Munc-Petersen (CAMP) factor gene (*cfb*), and the CylE deficient (Δ*cylE)* strain was done for 3 h (MOI 2.0). **(A)** IFNβ and TNF levels in the supernatant determined by ELISA (*n* = 4). **(B)** Cytokine transcript induction determined by quantitative real-time PCR (qPCR) (*n* = 6). **(C)** Infection of monocytes for 18 h with viable GBS wt (MOI 0.02-0.20-2.00) with or without TLR2 ligand FSL-1 (100 ng/ml) costimulation. Levels of IFNβ in the supernatants were determined with ELISA (*n* = 4), while IL-12-p70 levels were determined by bioplex (*n* = 3). In non-infected samples the levels were below the limit of detection (1 pg/ml for IFNβ and 20 pg/ml for IL-12-p70).

IFNβ induction by GBS in murine macrophages and human THP-1 cells is dependent on β-hemolytic activity, giving phagosomal damage and leakage of GBS DNA into the cytosol (26, 29). To examine the possible role of GBS hemolytic activity for cytokine induction in primary cells, we compared the GBS wt and GBS Δ*lgt* strains to isogenic strains deficient in β-hemolysin (Δ*cylE*) and cohemolysin/CAMP factor (Δ*cfb)*, as both these factors can contribute to phagosomal pore formation and the induction of IFNβ (26). The hemolysin-deficient phenotype was verified on blood agar (not shown). Infection of primary monocytes revealed that neither IFNβ nor TNF production was dependent on hemolysin (Figure 2A). On the contrary, at the transcriptional level, hemolysin deficiency slightly increased the IFNβ and TNF induction, suggesting that hemolysins to some extent can attenuate the cytokine responses (Figure 2B). The reasons for the tendency of hemolysin mutant strains to induce slightly higher cytokine transcript levels relative to wt bacteria are not entirely clear, but may be related to subtle hemolysin dependent cytotoxic effects.

To examine the effect of TLR2 signaling on cytokine induction by GBS, human primary monocytes were infected with viable GBS and with or without costimulation with the TLR2 ligand FSL-1. FSL-1 costimulation strongly reduced the production of IFNβ by both the GBS wt strain (Figure 2C) and the GBS Δ*lgt* (Figure S2A in Supplementary Material). Analysis of additional cytokines induced by the GBS wt strain revealed a similar effect of FSL-1 costimulation on IL-12-p70 production (Figure 2C), while FLS-1 had no effect the induction of TNF, IL-1β, and MCP-1, but elevated the levels of IL-6 and IL-10 (Figures S2B–F in Supplementary Material). We previously showed that the uptake of *S. aureus* in monocytes increases by FSL-1 costimulation, while the attenuation of IFNβ and IL-12 production is a result of cross-talk with TLR8-IRF5 signaling, and not a direct effect on the bacteria (24). The similar effects of FSL-1 costimulation on GBS-induced cytokine production indicate that sensing of also this bacterium might depend on endosomal TLR8-IRF5 signaling.

### GBS Strongly Activates IRF5

**Figure 3:**
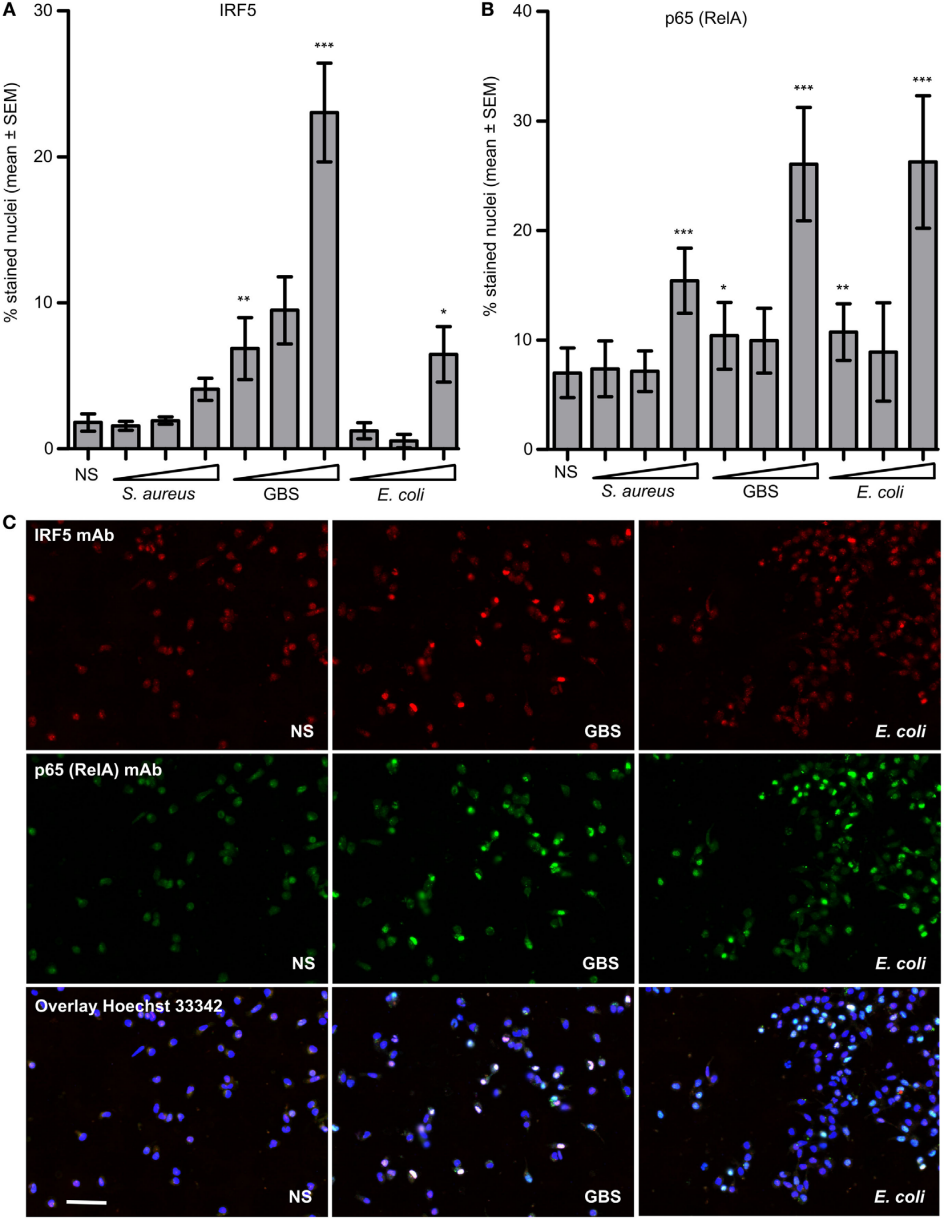
Group B *Streptococcus* (GBS) is a more potent activator of interferon regulatory factor 5 (IRF5) nuclear accumulation in monocytes than stationary grown *Staphylococcus aureus* and *Escherichia coli*. Monocytes were infected with viable *S. aureus* Cowan strain (MOI 0.20–0.64–2.00), GBS wt (MOI 0.10–0.32–1.00), or *E. coli* (MOI 0.20–0.64–2.00) for 2 h. Fixation and immunofluorescence (IF) double staining of IRF5 and NF-kB (p65/RelA) was subsequently performed, and quantification was done by high-content screening (Scan^R, 20×). **(A)** Percentage of nuclei positively stained for IRF5, and **(B)** p65. **(C)** Representative images of IRF5 and p65 staining in cell with no stimuli (NS), and GBS and *E. coli* infected monocyte cultures (scale bar 50 µm). Significance was tested relative to NS. *n* = 4 (*n* = 2 for the medium bacterial dose).

We infected monocytes with stationary grown *S. aureus*, GBS and *E. coli* and compared the nuclear accumulation of IRF5 and NF-kB subunit p65 (RelA). Nuclear accumulation of IRF5 was dependent on the bacterial dose and was considerably stronger with GBS compared to *E. coli* and *S. aureus* (Figure 3A). In contrast nuclear accumulation of p65 was induced to a similar extent by GBS and *E. coli* infection, but weaker by *S. aureus* (Figure 3B). The cells responding to GBS infection were mainly double-positive for IRF5 and p65 nuclear staining, while with *E. coli* only some cells showed nuclear IRF5 staining (Figure 3C). The differential activation of IRF5 with GBS and *S. aureus* correlated with the induction of IFNβ with these bacteria (Figure 1A), which suggests that TLR8-IRF5 signaling may be more important for sensing of GBS than stationary grown *S. aureus*. GBS infection did not trigger IRF3 phosphorylation, while this was clearly seen after LPS or *E. coli* stimulation (Figure S3 in Supplementary Material). This reflects the important role of IRF3 in TLR4-mediated induction of IFNβ from the endosome *via* the TRAM-TRIF-TBK1-IRF3 signaling pathway (30).

### Both TLR8 Signaling and Cytosolic DNA Sensing Activate IRF5

**Figure 4:**
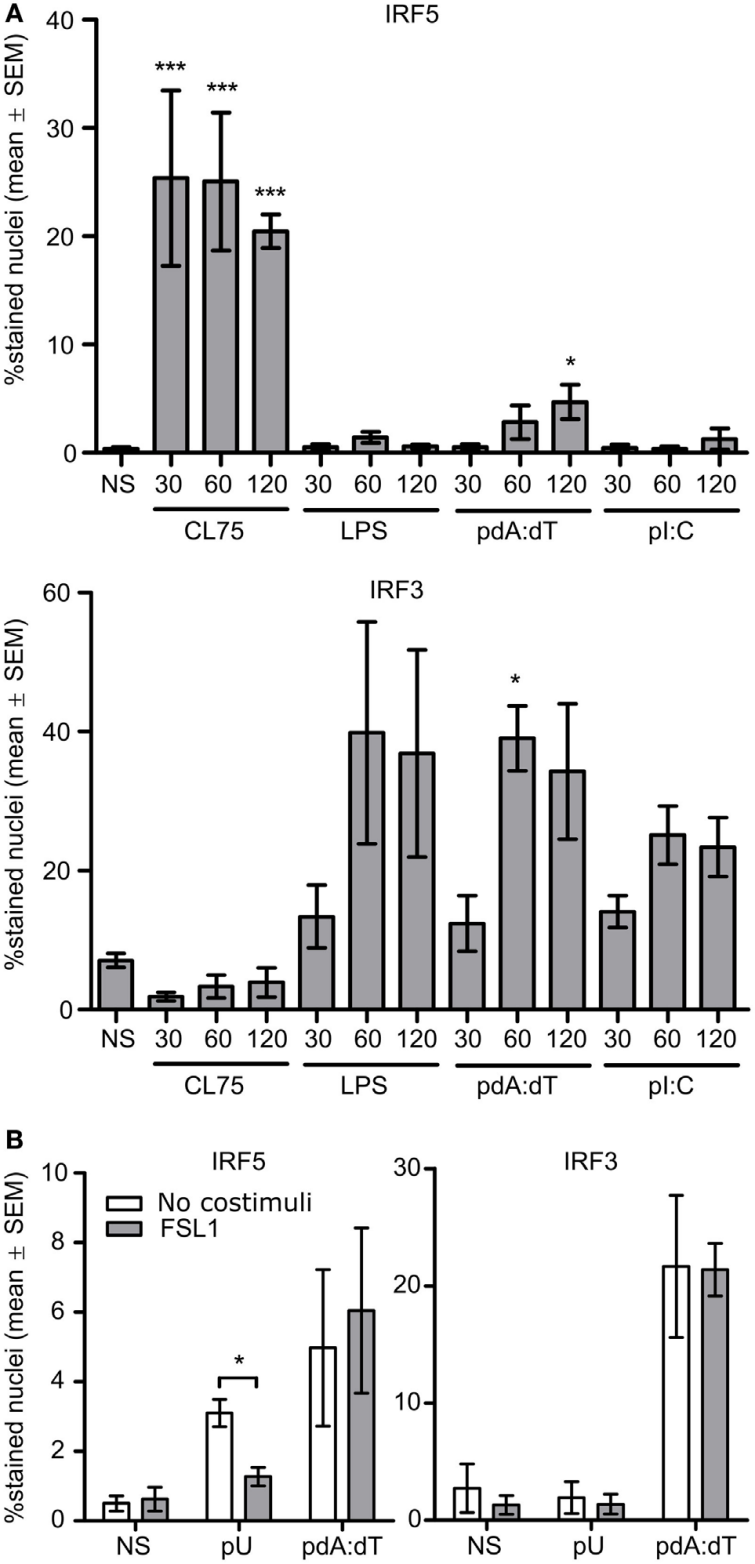
IFN regulatory factor 5 (IRF5) is activated by TLR8 ligands and cytosolic dsDNA sensing, IRF3 is activated by TLR4 signaling and cytosolic dsDNA and dsRNA sensors, while only TLR8 is antagonized by TLR2 ligand. **(A)** Monocytes were stimulated with CL75 (1 μg/ml), LPS (K12, 100 ng/ml), pdA:dT (dsDNA, 1 μg/ml), or poly(I:C) (dsRNA, 1 μg/ml). The nucleic acid ligands were transfected using L2K. Fixation was done at 30, 60, and 120 min poststimulation and IF staining of IRF5 and IRF3 was performed. **(B)** Monocytes were stimulated with the TLR8 agonist polyuridylic acid (pU) in complex with poly-l-arginine (pLA) or with pdA:dT/L2K. The ligands (1 μg/ml) were added alone or together with FLS-1 (100 ng/ml), and cells were fixed and stained after 120 min of incubation. Quantification of nuclear accumulation was done by high-content screening (Scan^R, 20×). Significance was tested relative to no stimuli (NS). *n* = 4.

To clarify the roles of IRF5 and IRF3 in IFNβ production by monocytes induced by distinct PRR ligands, monocytes were stimulated with the IFNβ inducing PRR ligands CL75, LPS, pdA:dT (dsDNA), and poly(I:C) (dsRNA). The nucleic acid ligands were premixed with Lipofectamine 2000 (L2K) for cytosolic transfection of the cargo, in contrast to premixing with cationic delivery agents such as pLA which gives endosomal localization (31). After 30, 60, and 120 min of treatment the cells were fixed and immunostained for IRF3 and IRF5 nuclear accumulation. Cytosolic transfection with pdA:dT gave a weak but significant IRF5 relocalization upon prolonged incubation, while no effect was seen with poly(I:C) transfection (Figure 4A). IRF5 was rapidly activated by the TLR8-ligand, but not at all by the TLR4-ligand (Figure 4A), in agreement with our previous study (24). Thus, in addition to the strong and rapid IRF5 relocalization by TLR8 signaling, cytosolic dsDNA sensing mechanisms can to some extent activate IRF5 nuclear translocation in human primary monocytes. In addition to LPS, TBK1-IRF3 activation is mediated *via* the cytosolic dsRNA sensing helicases melanoma differentiation-associated gene 5 (MDA5) and retinoic acid-inducible gene-I (RIG-I), and also the dsDNA sensors RNA polymerase III, DEAD box polypeptide 41 (DDX41), IFN inducible protein 16 (IFI16), DNA-dependent activator of IFN regulatory factors (DAI), and cyclic GMP-AMP synthase (cGAS) (32). Thus as expected both LPS and transfection of dsDNA and dsRNA strongly activated IRF3 translocation, while the TLR8 ligand did not. Moreover, IRF1 was not clearly activated by any of the ligands (Figure S4 in Supplementary Material), and neither was IRF7 activation seen (24). However we can not exclude the possibility that also other IRFs might be involved. We further examined the effect of TLR2-costimulation on IRF5 activation by the TLR8 ligand polyuridylic acid (pU) in complex with pLA, and IRF5 and IRF3 activation by cytosolic dsDNA (pdA:dT). While FSL-1 impaired IRF5 activation with pU/pLA, as well as pU/pLA mediated induction of both IFNβ (24) and IFNα (not shown), it influenced neither IRF5 nor IRF3 activation by cytosolic pdA:dT (Figure 4B). IFNβ induction by a TLR7 ligand in monocytes is not affected by FLS-1 costimulation (24), and the same holds true for different TLR7 ligands in human full blood, while a TLR9 ligand does not stimulate cells in whole blood or monocyte cultures (not shown). The TLR2-antagonistic effect thus seems to be restricted to TLR8-IRF5 activation in these model systems and does not inhibit IRF5 activation *via* other signaling pathways.

### TLR2 Signaling Antagonizes IRF5-Activation Induced by GBS, But Not by Viable *E. coli*

**Figure 5:**
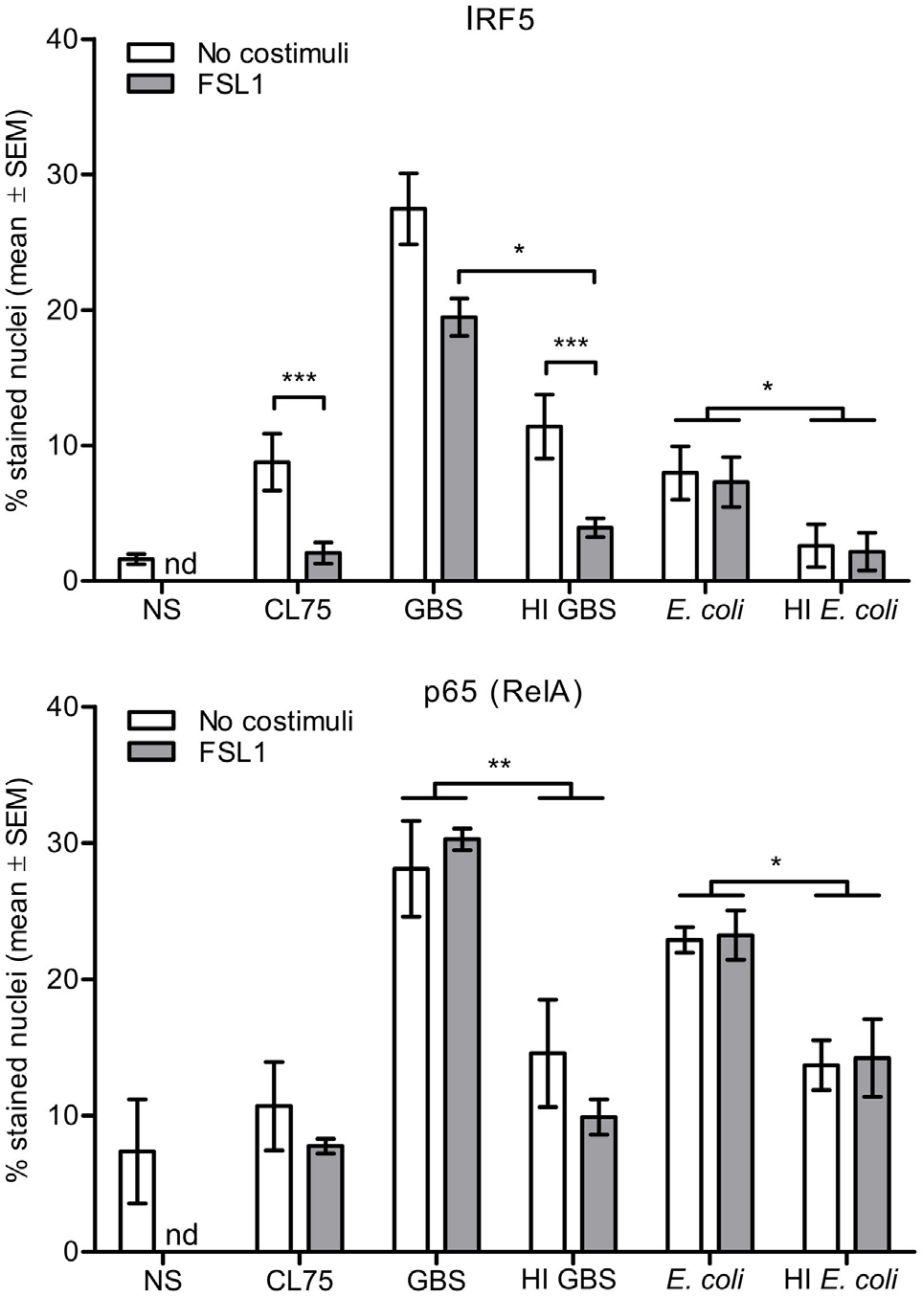
Activation of IFN regulatory factor 5 (IRF5) by TLR8 ligand and Group B *Streptococcus* (GBS) is antagonized by TLR2 costimulation, while activation of IRF5 by viable *Escherichia coli* is not. Monocytes were stimulated with CL75 (1 μg/ml), viable or heat-inactivated (HI) GBS (MOI 1.0) and *E. coli* (MOI 2.0), and with or without FSL-1 costimulation (100 ng/ml) for 2 h. The monocytes were subsequently fixed and double IF stained IRF5 and p65/RelA. Nuclear accumulation was quantified by high-content screening (Scan^R, 20×). NS, no stimuli. nd, not done. *n* = 4.

We further investigated the effect of TLR2 costimulation and HI on IRF5 and p65 nuclear translocation induced by GBS and *E. coli*. Human primary monocytes were infected with viable bacteria or stimulated with HI GBS, HI *E. coli* or the TLR8 ligand CL75, and nuclear staining was subsequently performed. No significant activation of IRF5 was seen with HI *E. coli*, while HI GBS activated IRF5 at a reduced level compared to viable GBS. The viable bacteria also activated p65 more potently than HI bacteria (Figure 5). The reduced potency of the HI GBS correlated with loss of RNA integrity (Figure S5 in Supplementary Material), suggesting that RNA is a vita-PAMP of GBS. This finding is similar to what has been described for *E. coli*, where inactivation by either heat or antibiotics resulted in rapid RNA degradation (33). Interestingly, while activation of IRF5 by TLR8 and GBS was reduced by TLR2 costimulation, IRF5 activation by *E. coli* was not affected by TLR2 ligand treatment. This suggests that *E. coli* activates IRF5 by a mechanism that is independent of TLR8 and possibly dependent on cytosolic nucleic acid sensing.

### TLR8 Is Recruited to GBS Phagosomes

**Figure 6:**
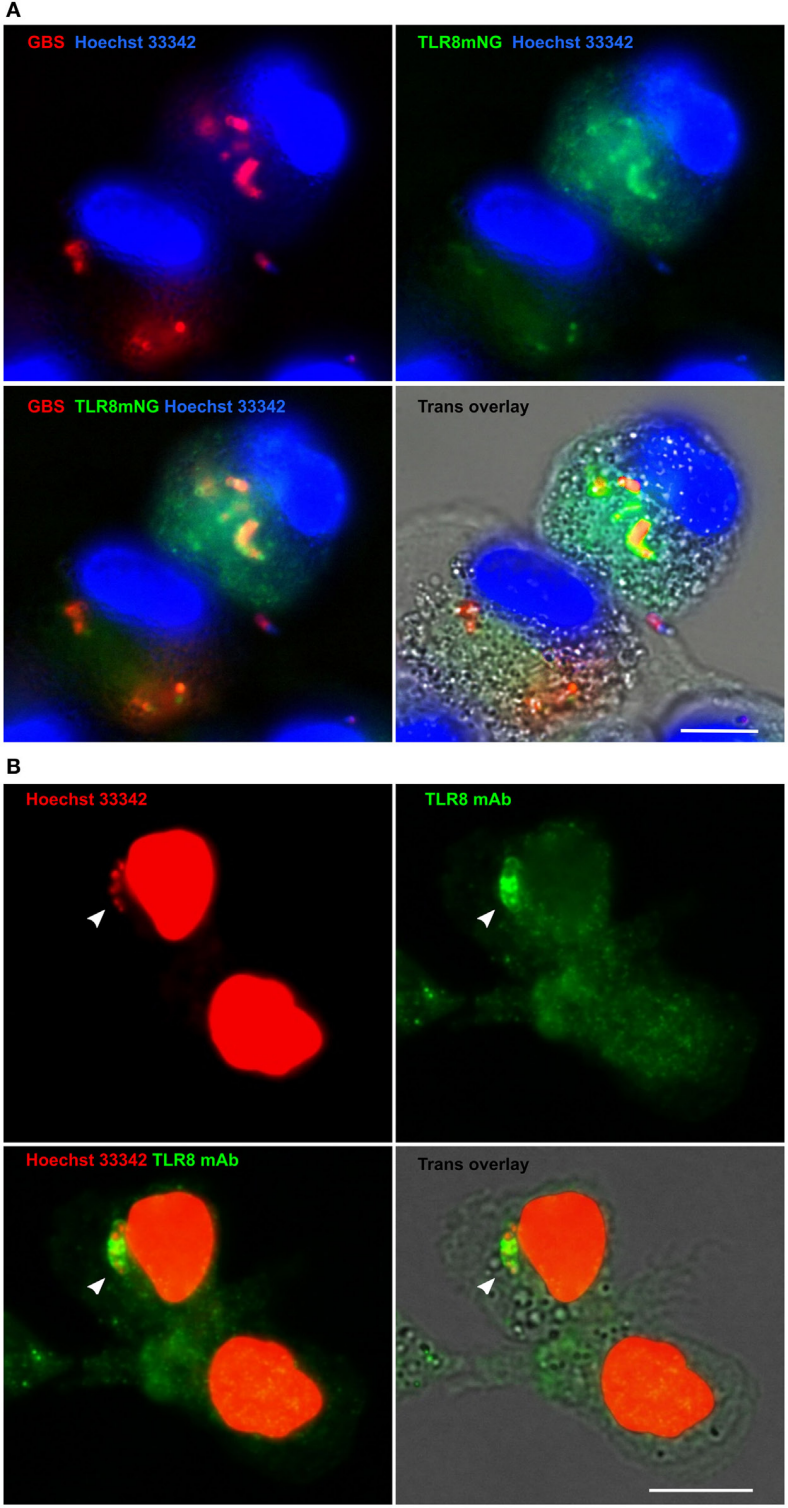
TLR8 accumulates on Group B *Streptococcus* (GBS) phagosomes in macrophages and monocytes. **(A)** THP-1 TLR8-KO cells were transduced with TLR8 mNeonGreen construct (TLR8mNG) and differentiated with PMA. Macrophages were infected with viable GBS (MOI 1.0) prestained with Alexa 647. Gentamycin was added 1 h postinfection and cells were fixed with paraformaldehyde after 20 h. DNA was stained with Hoechst 33342. **(B)** Primary monocytes were infected with viable GBS (MOI 10), and Gentamycin was added 1 h postinfection. Cells were fixed with paraformaldehyde after 3 h and TLR8 was IF stained with a mAb. DNA was stained with Hoechst 33342 in PBS/saponin to visualize GBS phagosomes (arrowhead) and monocyte nuclei. Images were acquired using Olympus Scan^R (60×). Scale bars are 10 µm.

Several lines of evidence suggest that GBS is sensed by TLR8 in human primary monocytes. To examine whether TLR8 is recruited to GBS phagosomes, we overexpressed TLR8 in THP-1 cells. Using TLR8 knockout cells (19), we overexpressed a TLR8 mNeonGreen construct (TLR8mNG). The cells were differentiated to macrophages with PMA and infected with viable GBS. Imaging of fixed cells revealed accumulation of TLR8 on GBS phagosomes in roughly one half of the cells with medium level of fluorescent TLR8 expression (Figure 6A). We also examined TLR8 localization in primary monocytes by IF. The specificity of TLR8 immunostaining was tested using THP-1 cells overexpressing full length TLR8 (Figures S6A,B in Supplementary Material). Staining of primary monocytes infected with viable GBS revealed accumulation of endogenous TLR8 on phagosomes (Figure 6B), similar to the findings with the TLR8-mNG recruitment in THP-1. We also examined recruitment of TLR9 in TLR9-mNG expressing THP-1 cells, but TLR9 was mainly localized to the endoplasmatic reticulum and did not accumulate on GBS (not shown). IF of TLR4 in primary monocytes did not reveal any TLR4 recruitment to *S. aureus* or GBS phagosomes, while *E. coli* phagosomes were clearly stained (Figure S6C in Supplementary Material). These results are in agreement with our previous study using HI bacteria (30).

### Role of TLR7, TLR8, IRF5, and STING in the Sensing of GBS and *E. coli*

**Figure 7:**
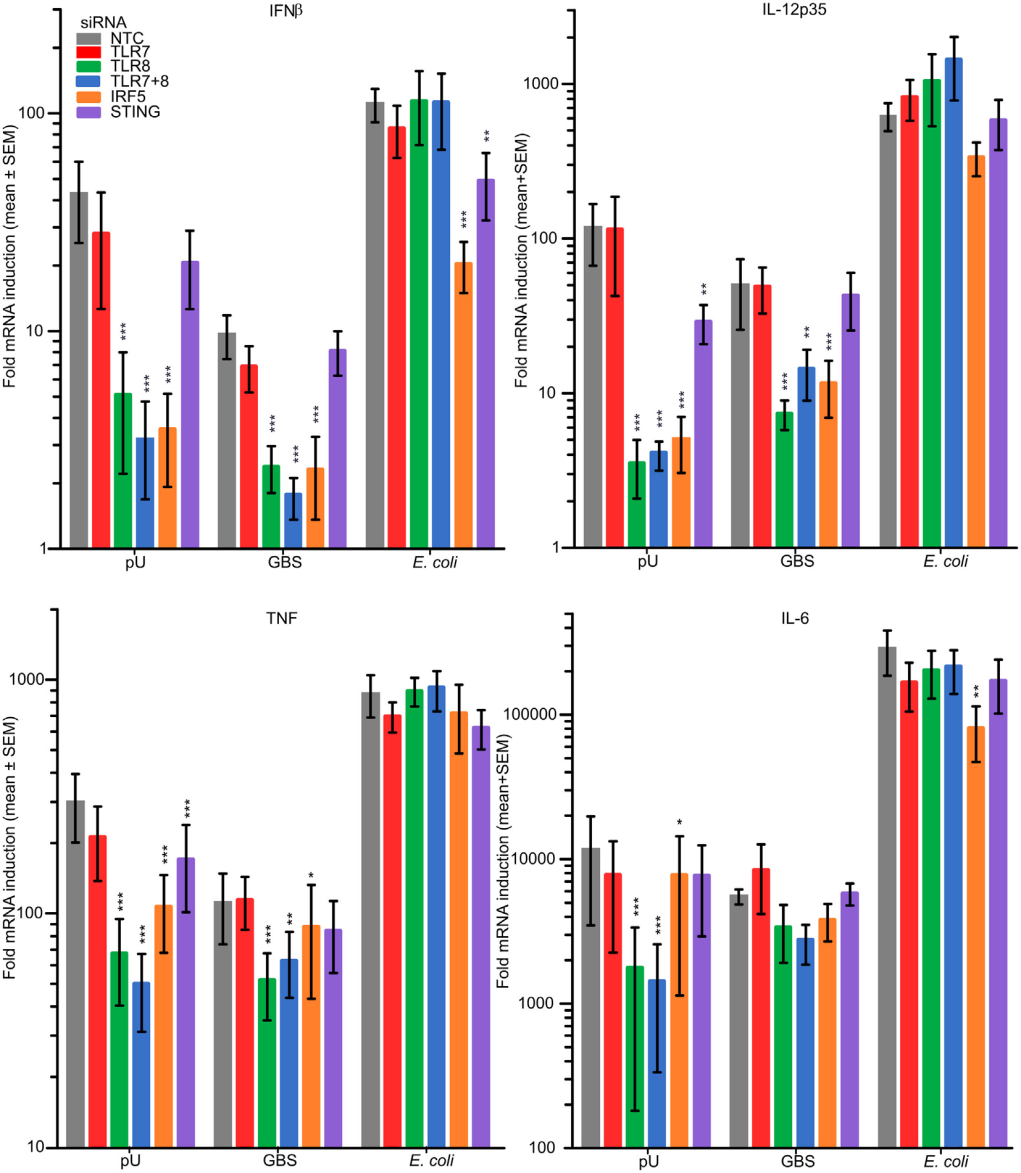
Group B *Streptococcus* (GBS) trigger cytokine production in part *via* TLR8-IRF5 signaling, while *Escherichia coli* induces cytokines independently of TLR8, yet partly dependent on IRF5. Monocyte-derived macrophages (MDM) were transfected with siRNA against TLR7, TLR8, IRF5, and STING, as indicated. Following successful silencing the cells were stimulated with pU/pLA or infected with viable GBS wt (MOI 1.0) or *E. coli* (MOI 2.0). Cytokine induction relative to non-stimulated cells after 4 h of infection was determined by quantitative real-time PCR (qPCR). Significance of gene silencing was tested in relation to non-targeting control (NTC). *n* = 5.

To determine the role of TLR7, TLR8, IRF5, and STING (stimulator of interferon genes) for cytokine induction in MDM upon infection with viable GBS and *E. coli*, we performed siRNA-mediated gene silencing, which was efficient for all targets examined (Figure S7A in Supplementary Material) The pU/pLA induced cytokines were strongly reduced after TLR8 silencing (Figure 7). TLR7 knockdown did not significantly affect cytokine induction by either TLR8 ligand or bacteria. In contrast, silencing of TLR8 and IRF5 strongly impaired the GBS activated induction of IFNβ and IL-12-p35, and to some degree also TNF (Figure 7). IL-6 induction by GBS was not significantly affected by TLR8 or IRF5 knockdown, suggesting that TLR8 is redundant with other PRRs for the induction of this particular cytokine. Silencing IRF5 also affected pU-mediated induction of IL-6 less efficiently compared to the other cytokines and suggests that IL-6 transcription is mainly dependent on the more general factors NF-kB and MAPKs. None of the GBS induced cytokines was STING dependent (Figure 7). Induction of cytokines by *E. coli* was independent of TLR8, but maximal IFNβ and IL-6 induction was partly dependent on IRF5. A TLR8-independent mechanism of IRF5 activation by viable *E. coli* is supported by the nuclear translocation studies, because this was insensitive to TLR2 costimulation (Figure 5). Moreover, a role of STING for the induction of IFNβ by *E. coli* is indicated, suggesting the possible involvement of a STING-IRF5 signaling axis (Figure 7). STING can be activated by cyclic dinucleotides produced by cGAS upon cytosolic DNA sensing, or by cyclic dinucleotides produced by the bacteria (34). Silencing of cGAS in MDM did not attenuate *E. coli*-mediated induction of IFNβ (Figure S7B in Supplementary Material), suggesting that STING is not activated by viable *E. coli via* the DNA-cGAS-cGAMP (cyclic-di-GMP-AMP) pathway. MyD88 or IKKβ knockdown also did not inhibit IFNβ induction by *E. coli* (Figure S7B in Supplementary Material), which supports that endosomal TLR signaling is not involved (Figure 7). TBK1 silencing, on the other hand, attenuated the *E. coli*-mediated IFNβ production (Figure S7B in Supplementary Material), which is expected given its central role in the TLR4 signaling pathway upstream of IRF3 (30).

In conclusion, TLR8-IRF5 signaling is a major contributor to the sensing of GBS in primary human monocytes and MDM by inducing IFNβ, IL-12, and TNF. In comparison with GBS, TLR8 is less strongly activated by stationary grown *S. aureus* and does not contribute to cytokine induction by viable or HI *E. coli*. Still, IRF5 plays a role in cytokine induction by viable *E. coli*.

## Discussion

We here identify TLR8 as a sensor of GBS in human monocytes and MDM which is essential for the induction of the cytokines IFNβ and IL-12, and which also contributes to TNF production. Induction of these cytokines was dependent on IRF5, and IRF5 activation could be antagonized by simultaneous TLR2 signaling. GBS RNA is likely the TLR8-activating ligand, and TLR8 can recognize isolated RNA of different bacterial species including streptococci (25). Also, the attenuated IRF5 activation by HI GBS correlates with loss of bacterial RNA integrity.

TLR13 is also an endosomal sensor of RNA of various bacteria, including streptococci, but in contrast to TLR8 it is highly specific for a 23S rRNA motif, is sensitive to specific RNA methylations, and is not expressed in murine monocytes (13, 25). Thus, while TLR8 and TLR13 are endosomal sensors of ssRNA, they differ in several important aspects. The highly restricted specificity of TLR13 is makes it vulnerable to microbial immune evasion *via* rRNA modifications, while the broad specificity of TLR8 might be a risk factor for development of autoinflammatory reactions upon sensing of endogenous ssRNA (19).

The stronger TLR8-IRF5 activation by GBS relative to *S. aureus* from stationary growth results in enhanced cytokine induction, in particular IFNβ and IL-12. Type I IFN may have a key part in the dysregulated inflammatory condition that characterizes bacterial sepsis, and can stimulate or suppress immune responses depending on the bacterial species and route of infection (35, 36). In murine infection models type I IFN are generally protective against GBS and other streptococci *via* suppression of excessive inflammation, promotion of tissue integrity, or by unknown mechanisms, while for infections with other bacterial species type I IFN are often associated with detrimental effects (36). Thus, TLR8 and type I IFN might play a protective role in sepsis caused by GBS and other streptococci. In mice, however, type I IFN may inhibit DC functions leading to T-cell paralysis (37). This raises the question of how innate immunity and TLR8 regulate adaptive immunity against GBS and other extracellular bacteria. Generally, both the cytokine environment and PRR signaling can regulate antigen presentation and Th-cell development. We have revealed a TLR8-IRF5 signaling mechanism inducing IFNβ and IL-12 under the regulation of TLR2. In mouse IRF5 is a marker of M1 macrophages which produce IL-12 supporting the development of Th1 and Th17 responses (38). A TLR7 ligand used as adjuvant enhanced the antibody titers and the Th1 and Th17 responses toward *S. aureus* in mice, thus improving the protection (39). Bacterial RNA has also an adjuvant effect enhancing the antibody titers against *E. coli* (33). Moreover, the dual TLR7/8 agonist Resiquimod modulated human monocyte-derived DC differentiation which promoted T-cell proliferation (40). Deficiency in NEMO and IkBa give a severe immunological phenotype in humans which includes impaired antibody responses, while MyD88 and IRAK-4 deficiency results in a less severe phenotype with normal B- and T-cell responses, though in some patients polysaccharide specific responses are reduced (41). These findings suggest that some central components of innate immunity are essential for adaptive immunity, while TLR- and/or IL-1R-signaling may be less important, but can promote antibody production in at least some humans. TLR8 induced cytokines such as type I IFN and IL-12 might play a role here.

Gene silencing of TLR7 did not affect GBS- or *E. coli*-mediated induction of cytokines. This is in agreement with our previous study on *S. aureus*, as well as the poor effect of TLR7 ligands on monocytes compared to TLR8 ligands (17, 31). GBS-mediated induction of IFNβ in THP-1 cells is exclusively dependent on β-hemolysin mediating leakage of DNA into cytosol and sensing *via* the cGAS-STING pathway (29). We could not reveal such a mechanism for cytokine induction by GBS in our study through silencing of STING or by using GBS strains deficient in hemolysin, and we argue that TLR8 is more important for GBS sensing in primary phagocytes. On the other hand, a possible role of STING silencing was indicated for the induction of IFNβ and IL-6 by viable *E. coli*.

Differential IFNβ induction by GBS and *S. aureus*, as found with monocytes and MDM, has also been observed in a study using murine DC. Here, poor responses to stationary phase *S. aureus* was attributed to its highly lysozyme resistant peptidoglycan which limits bacterial degradation in phagolysosomes, while IFNβ was induced *via* a TLR-independent RNA-sensing mechanism (42). A more easily degradable cell wall of GBS thus likely in part explains why this bacterium activates TLR8 more efficiently than stationary phase *S. aureus*. In the current and previous studies, we revealed a major reduction in TLR8 activation by *S. aureus* as the bacterium enters the stationary growth phase. While bacterial viability was sustained for at least 24 h, various physiological changes occur to *S. aureus* in this phase, including increased cell-wall thickness, reduced size, reduced respiratory activity, and reduced protein content (43). We believe increased cell wall thickness and reduced amount of bacterial RNA can be two mechanisms limiting TLR8 activation by stationary phase *S. aureus*, and in addition we have revealed increased accumulation of secreted TLR2 ligands by various *S. aureus* strains during growth (24). In comparison, the production of TLR2-activating lipoproteins by most GBS strains is limited (7) and was insufficient in attenuating TLR8 activation in our study.

TLR8 seems redundant with other PRRs for IL-6 induction by GBS, which is in contrast to findings with *S. pyogenes* where both IL-6 and IFNβ was induced in a TLR8-dependent fashion (25). The reason for this discrepancy is unclear, and incomplete silencing of TLR8 on the protein level may be one explanation. Also, IL-6 induction by TLR8 ligand seems IRF5 independent, and large redundancy among PRRs can be expected for a cytokine induced mainly *via* MAPKs and NF-kB.

Using THP-1 wt and TLR8 knockout cells, we were unable to reveal a TLR8-mediated response to bacteria, which is in contrast to a previous study that showed attenuated cytokine production to viable *S. aureus* and *E. coli* in this TLR8 knockout line (19). The discrepancy might be related to an unstable phenotype of THP-1 sub-clones during cultivation and freezing, as the TLR8 knockout line also gave an attenuated response to purified LPS (data not shown). Moreover, THP-1 cells may be less efficient in bacterial phagocytosis and degradation compared to primary phagocytes, and they have a relative low response to synthetic TLR8-ligands. Yet another notable difference of these cell types is the dependency on hemolysin for induction of cytokines by GBS in THP-1 cells but not primary monocytes. This THP-1 specific sensing mechanisms might obscure a TLR8-response to GBS. We could neither reveal a role of TLR8 for the sensing of *E. coli* in primary myeloid cells. RNA methylation in *E. coli* can block the activation of TLR13 but not TLR8, and TLR8 can indeed sense *E. coli* RNA (19, 25). TLR4 activation by *E. coli* LPS does not attenuate TLR8-IRF5 activation (24), though TLR2 and TLR5 ligands produced by *E. coli* might do so. We believe a possible weak activation of TLR8 by *E. coli* would be camouflaged by the more potent responses induced by TLR4 and additional PRR. One may also speculate that *E. coli* and the bacterial RNA gets degraded too rapidly to elicit an efficient TLR8 response, or that distinct subcellular localization and sorting of bacteria and sensors play a regulatory role.

*E. coli* mRNA was identified as a *vita*-PAMP in mouse macrophages where leakage of intact RNA into the cytosol activated both IFNβ and IL-1β production *via* a TRIF, NLRP3, and caspase-1 dependent mechanism (33). The nature of the presumed *E. coli vita*-PAMPs was not identified in the current study, but RNA leaking into the cytosol is one candidate. *E. coli* RNA might activate IRF5 *via* RIG-I or MDA5 which are expressed in human monocytes (31). However, as IRF5 was activated by transfection of pdA:dT but not pI:C, we cannot exclude that cytosolic sensing of *E. coli* DNA independent of cGAS is involved. A clarification of these molecular mechanisms will require further investigations.

We conclude that TLR8 is an important PRR of GBS in human primary myeloid phagocytes. In comparison, *S. aureus* from stationary growth phase less strongly activates TLR8, while for sensing of *E. coli* TLR8 is not a major contributor.

## Ethics Statement

Buffycoats were acquired from healthy volunteers under informed written consent approved by the Regional Committee for Medical and Health Research Ethics (REC Central, Norway, no. 2009/2245).

## Author Contributions

TE, GT, JD, BE, and JS contributed to the conception and design of the work. KB made the TLR8-mNG overexpressing THP-1 cells and did the confocal imaging. MY made TLR8 (wt) overexpressing THP-1 cells, did the TLR8 Western blot, and the TLR4 IF labeling. BE conducted infection experiments, cytokine analyses, and siRNA experiments together with SM, JK, and JS. SM also performed THP-1 experiments and did the IRF3 Western blot. GL did the GBS RNA isolation together with BE and assisted with GBS mutants. Other IF experiments and all Scan^R analyses was done by JS. BE and JS wrote the manuscript with critical revisions by the other authors. All authors approved the final manuscript version.

## Conflict of Interest Statement

The authors declare that the research was conducted in the absence of any commercial or financial relationships that could be construed as a potential conflict of interest.

## Supplementary Material

Figure S1 Influence of the bacterial growth phase and culture supernatant on induction of IFNβ and TNF. Human primary monocytes (Mo) were infected with viable GBS wt, GBS Δ*lgt* (MOI 2.0), *S. aureus* wt, and *S. aureus* Δ*lgt* (MOI 4.0) from exponential growth phase or stationary phase, and for stationary phase with or without removal of the bacterial culture media. Cytokine induction was determined by qPCR 3 h post infection (*n* = 2–4). The figure is related to Figure 1.

Figure S2 Effect of FSL-1 costimulation on GBS-induced cytokines in primary monocytes. Infection of monocytes for 18 h with viable GBS Δ*lgt* **(A)** or GBS wt **(B–F)** (MOI 0.02-0.20-2.00) with or without TLR2 ligand FSL-1 (100 ng/ml) costimulation. Levels of IFNβ in the supernatants were determined with ELISA (*n* = 4), while the other cytokines were determined by bioplex (*n* = 3). The figure is related to Figure 2.

Figure S3 Analysis of IRF3 phosphorylation in monocytes after stimulation with LPS or infection by *E. coli* or GBS. Primary monocytes were stimulated with smooth LPS (100 ng/ml) and infected with *E. coli* or GBS (MOI 2.0) for the indicated time. Cells were lysed and the level of phosphorylated IRF3 and total GAPDH were analyzed by Western blot (kDa size marker indicated). The figure is related to Figure 3.

Figure S4 Quantification of IRF1 nuclear staining of monocytes after stimulation with PRR ligands. Primary monocytes were stimulated with CL75 (1 μg/ml) and LPS (K12, 100 ng/ml), or 1 μg/ml pdA:dT or polyI:C transfected with L2K. Fixation was done at 30, 60, and 120 min post stimulation, and IF staining of IRF1 was performed. Quantification was done by high-content screening (Scan^R, 20×). Significance levels are tested to no stimuli (NS) or as indicated. *n* = 4. The figure is related to Figure 4.

Figure S5 Characterization of RNA from viable and HI GBS. RNA was isolated as described in triplicates, and the integrity was examined on a bioanalyzer. RNA fragment ladder and corresponding nucleotide (nt) fragment lengths are indicated. Only RNA isolated from viable GBS show the 23S and 16S rRNA bands, as RNA from HI GBS is degraded. The RNA concentrations in the samples were on average 39 and 15 μg/ml for the viable and HI GBS, respectively. The figure is related to Figure 5.

Figure S6 IF staining of TLR8 in THP-1 cells and TLR4 in primary monocytes. THP-1 (wt) monocytes were transduced with empty vector (pDest) or vector with TLR8 (pDest-TLR8) and differentiated with PMA for 3 days. **(A)** THP-1 macrophages were stained with anti TLR8 mAb (XP, CST). A representative image was captured with Olympus Scan^R (20×). Scale bar is 10 µm. **(B)** Western blot of TLR8 and GAPDH in THP-1 lysate (kDa size marker indicated). **(C)** Human primary monocytes were infected with *S. aureus*, GBS or *E. coli* for 2 h and fixed, IF stained using TLR4 antibody (H80, Santa Cruz), and imaged using confocal microscopy. No accumulation of TLR4 was observed on *S. aureus* or GBS phagosomes, while clear accumulation of TLR4 was observed on *E. coli* phagosomes. Representative maximum intensity projection images of at least 10 cells per condition are shown. Scale bar is 5 µm. The figure is related to Figure 6.

Figure S7 Efficiency and effect of gene silencing in MDM. **(A)** The level of expression of various targets after siRNA-mediated silencing relative to a non-targeting control (NTC) was determined by RT-qPCR (*n* = 2–4). **(B)** Following successful gene silencing (right figure, *n* = 4) MDM were infected with *E. coli* (MOI 2.0). IFNβ induction after 4 h of infection relative to non-stimulated cells was determined by qPCR, and the significance of gene silencing on IFNβ expression was tested against non-targeting control (NTC) (*n* = 9). The figure is related to Figure 7.

## Abbreviations

cGAS, cyclic GMP-AMP synthase; GBS, Group B *Streptococcus*; HI, heat inactivated; IF, immunofluorescence; IKK, IkB kinase; IRF, IFN regulatory factor; *lgt*, prolipoprotein diacylglyceryl transferase gene; L2K, Lipofectamine 2000; MDA5, melanoma differentiation-associated gene 5; MDM, monocyte-derived macrophage; MyD88, myeloid differentiation factor 88; PAMP, pathogen-associated molecular pattern; pdA:dT, poly(deoxyadenylic-deoxythymidylic acid); pLA, poly-l-arginine; PMA, phorbol 12-myristate 13-acetate; poly(I:C), polyinosinic-polycytidylic acid; PRR, pattern recognition receptor; pU, polyuridylic acid; qPCR, quantitative real-time PCR; RIG-I, retinoic acid-inducible gene-I siRNA, small interfering RNA; STING, stimulator of IFN genes; wt, wild-type.
