# Supplementary figures and images for: Toll-Like Receptor 8 Is a Major Sensor of Group B Streptococcus But Not Escherichia coli in Human Primary Monocytes and Macrophages
Source: Front Immunol. 2017 Oct 3;8:1243. doi: 10.3389/fimmu.2017.01243 (PMC5632357; doi:10.3389/fimmu.2017.01243)

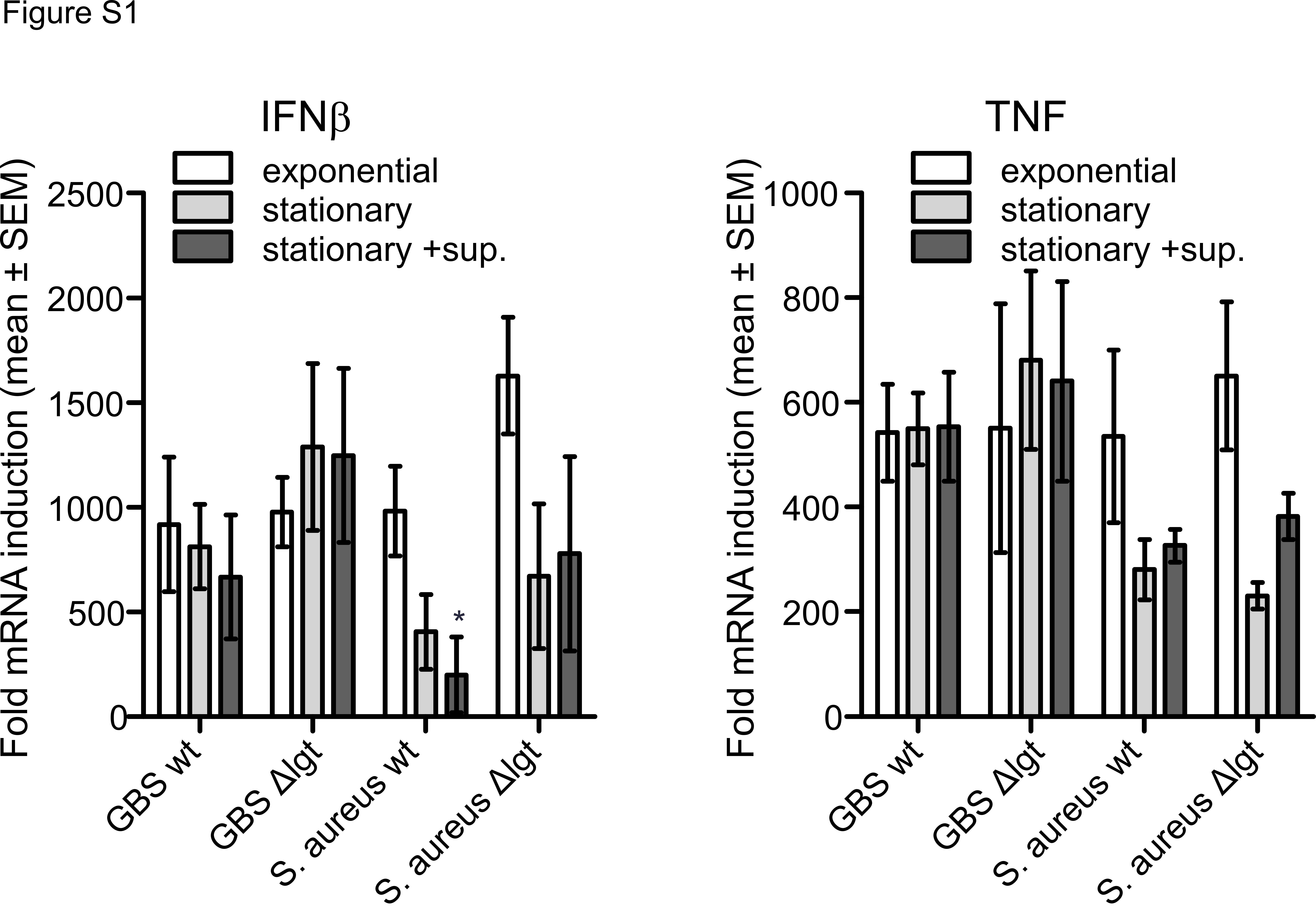

Supplement: Figure S1 — Influence of the bacterial growth phase and culture supernatant on induction of IFNβ and TNF. Human primary monocytes (Mo) were infected with viable GBS wt, GBS Δlgt (MOI 2.0), S. aureus wt, and S. aureus Δlgt (MOI 4.0) from exponential growth phase or stationary phase, and for stationary phase with or without removal of the bacterial culture media. Cytokine induction was determined by qPCR 3 h post infection (n = 2–4). The figure is related to Figure 1. [file image_1.tif]

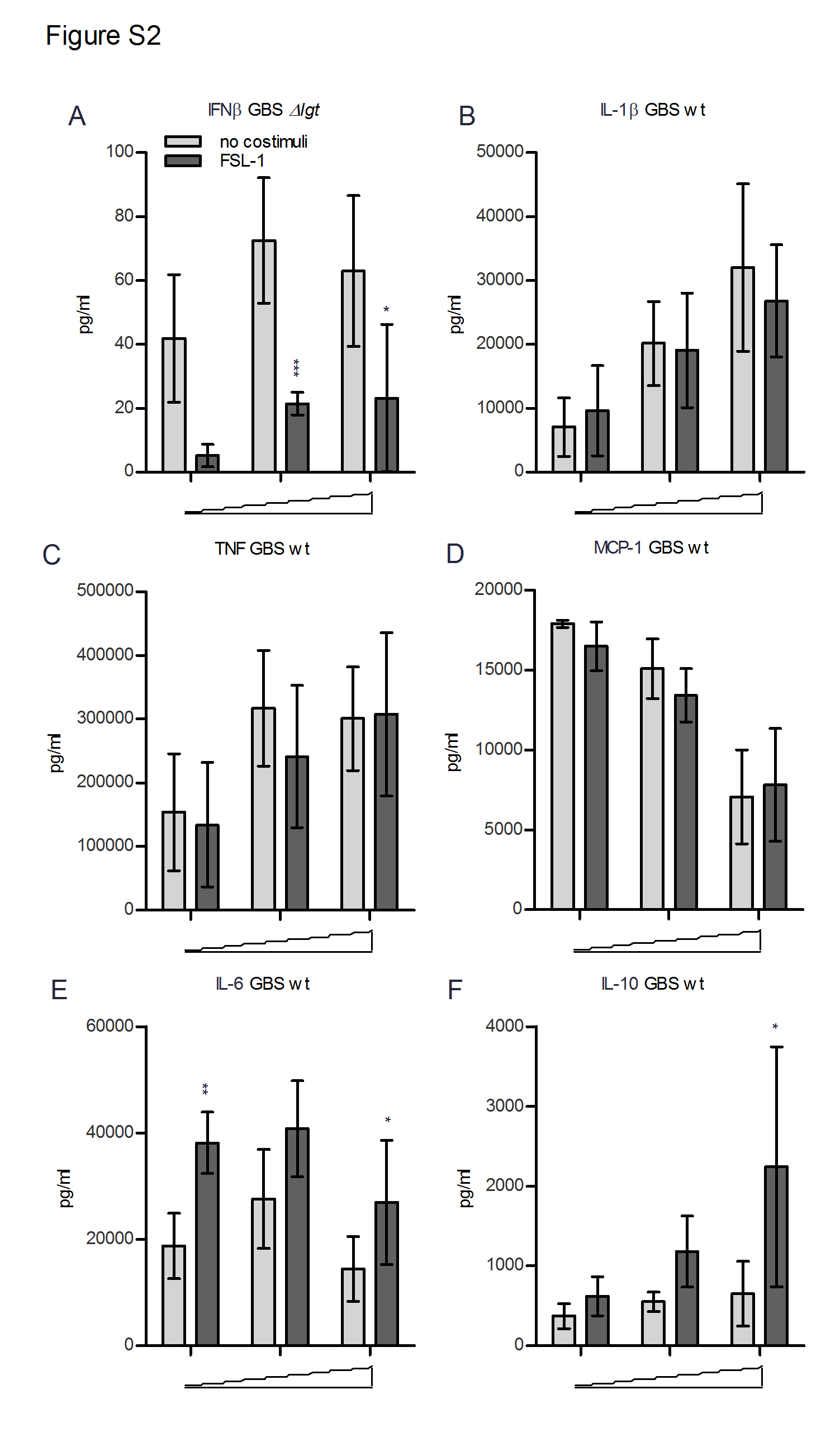

Supplement: Figure S2 — Effect of FSL-1 costimulation on GBS-induced cytokines in primary monocytes. Infection of monocytes for 18 h with viable GBS Δlgt (A) or GBS wt (B–F) (MOI 0.02-0.20-2.00) with or without TLR2 ligand FSL-1 (100 ng/ml) costimulation. Levels of IFNβ in the supernatants were determined with ELISA (n = 4), while the other cytokines were determined by bioplex (n = 3). The figure is related to Figure 2. [file image_2.tif]

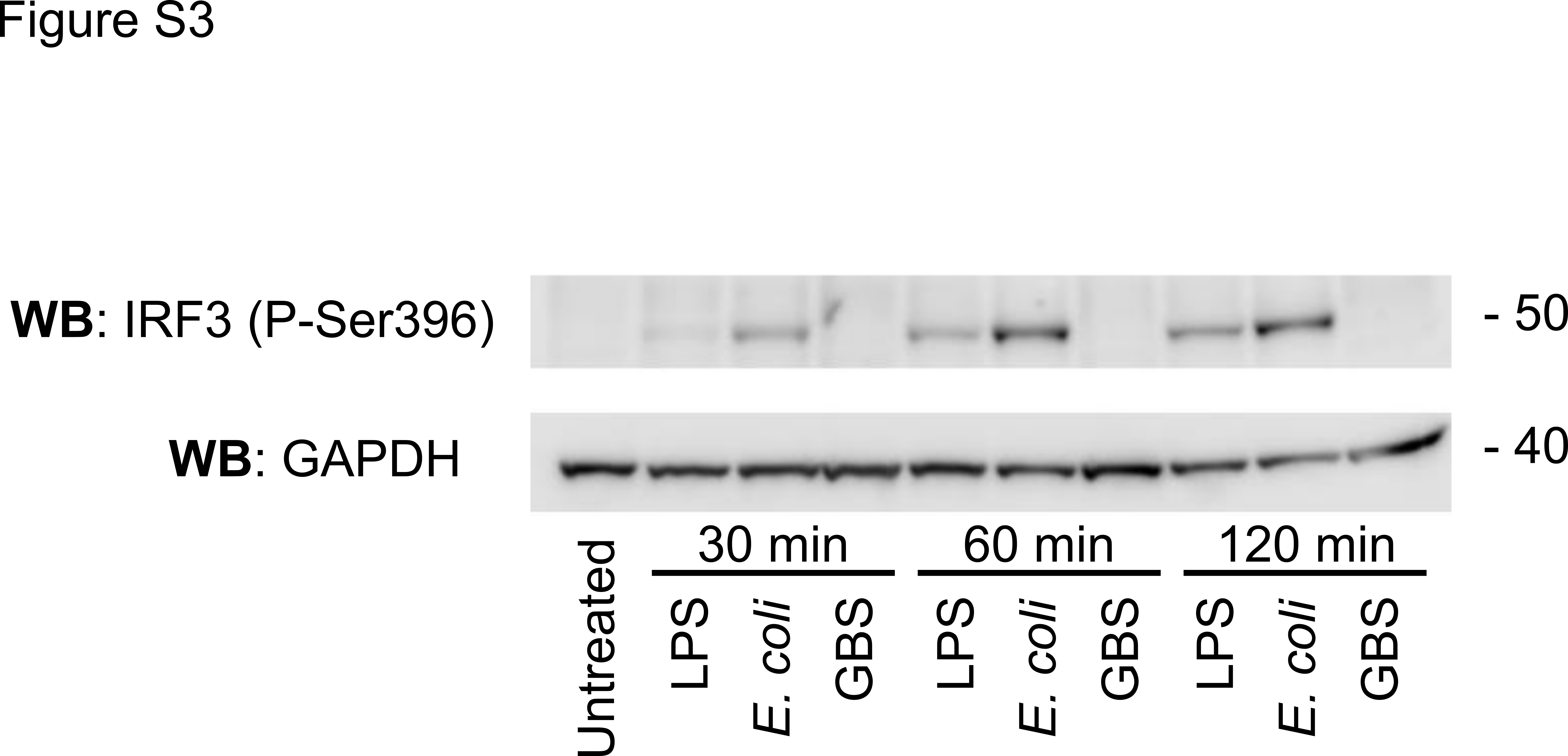

Supplement: Figure S3 — Analysis of IRF3 phosphorylation in monocytes after stimulation with LPS or infection by E. coli or GBS. Primary monocytes were stimulated with smooth LPS (100 ng/ml) and infected with E. coli or GBS (MOI 2.0) for the indicated time. Cells were lysed and the level of phosphorylated IRF3 and total GAPDH were analyzed by Western blot (kDa size marker indicated). The figure is related to Figure 3. [file image_3.tif]

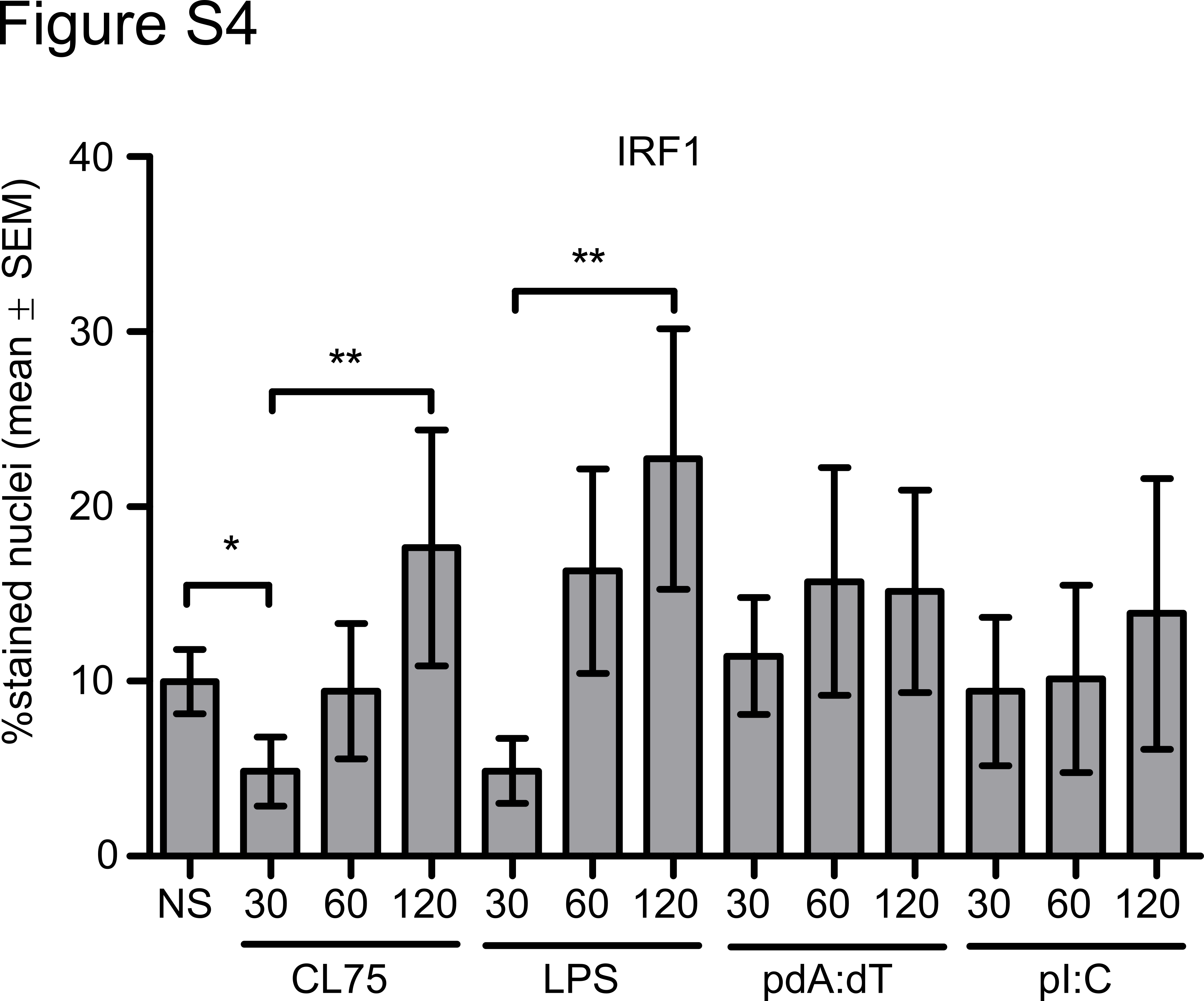

Supplement: Figure S4 — Quantification of IRF1 nuclear staining of monocytes after stimulation with PRR ligands. Primary monocytes were stimulated with CL75 (1 μg/ml) and LPS (K12, 100 ng/ml), or 1 μg/ml pdA:dT or polyI:C transfected with L2K. Fixation was done at 30, 60, and 120 min post stimulation, and IF staining of IRF1 was performed. Quantification was done by high-content screening (Scan^R, 20×). Significance levels are tested to no stimuli (NS) or as indicated. n = 4. The figure is related to Figure 4. [file image_4.tif]

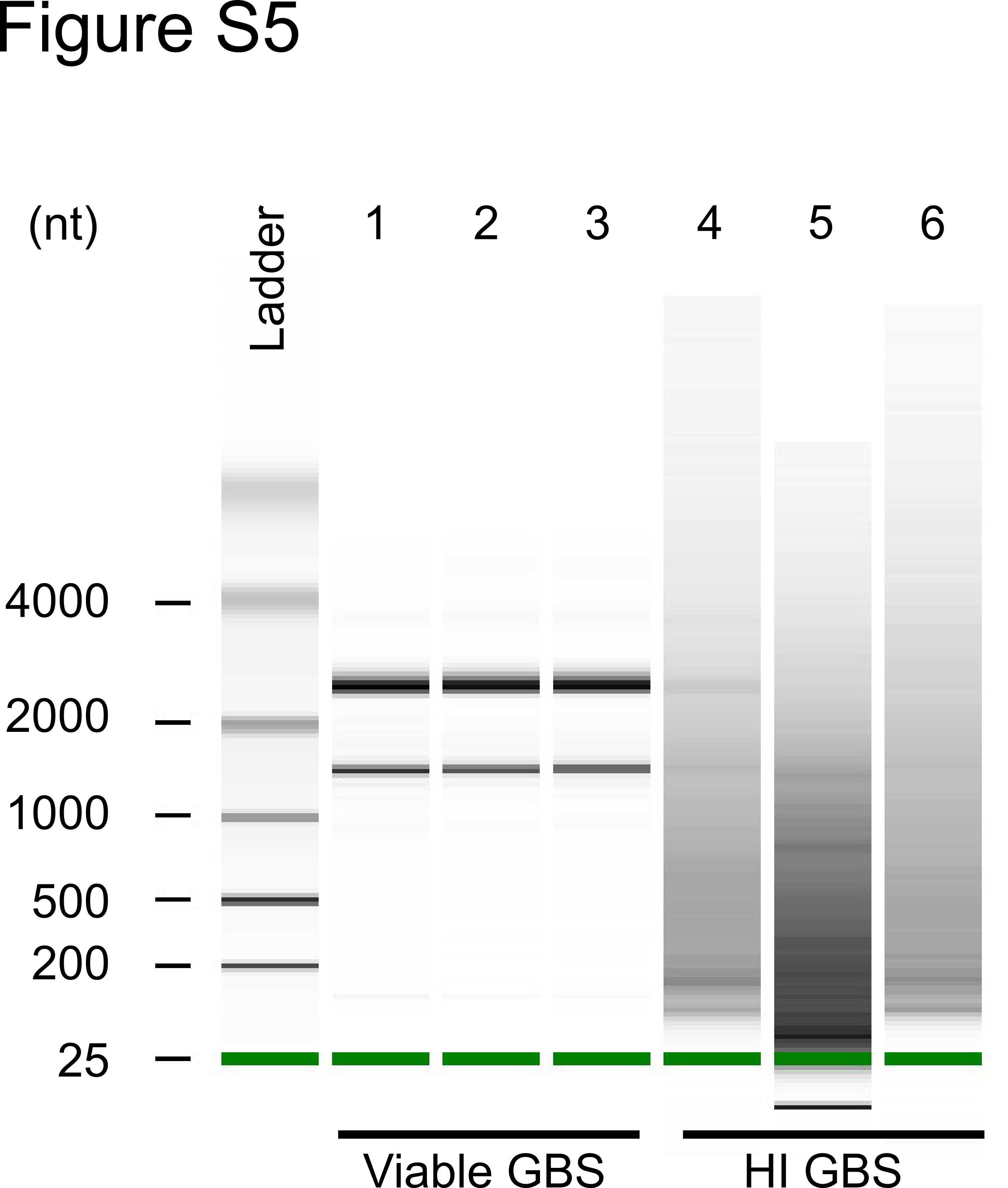

Supplement: Figure S5 — Characterization of RNA from viable and HI GBS. RNA was isolated as described in triplicates, and the integrity was examined on a bioanalyzer. RNA fragment ladder and corresponding nucleotide (nt) fragment lengths are indicated. Only RNA isolated from viable GBS show the 23S and 16S rRNA bands, as RNA from HI GBS is degraded. The RNA concentrations in the samples were on average 39 and 15 μg/ml for the viable and HI GBS, respectively. The figure is related to Figure 5. [file image_5.tif]
